# Correspondence: Reply to ‘Challenges with dating weathering products to unravel ancient landscapes’

**DOI:** 10.1038/s41467-017-01468-6

**Published:** 2017-11-15

**Authors:** Ola Fredin, Giulio Viola, Horst Zwingmann, Ronald Sørlie, Marco Brönner, Jan-Erik Lie, Else Margrethe Grandal, Axel Müller, Annina Margreth, Christoph Vogt, Jochen Knies

**Affiliations:** 1 Geological Survey of Norway, Leiv Eirikssons vei 39, 7491 Trondheim, Norway; 2 Department of Geography, Norwegian University of Science and Technology, 7491 Trondheim, Norway; 3 Department of Biological, Geological and Environmental Sciences–BiGeA, University of Bologna, 40126 Bologna, Italy; 4 Department of Geology and Mineralogy, Kyoto University, Kitashirakawa Oiwake-cho, Kyoto, 606-8502 Japan; 5 Lundin Petroleum AS, 1366 Lysaker, Norway; 6 Department of Petroleum Engineering and Applied Geophysics, Norwegian University of Science and Technology, 7491 Trondheim, Norway; 7 Natural History Museum, University of Oslo, 0318 Oslo, Norway; 8 Natural History Museum, London, SW7 5DB UK; 9 ZEKAM/FB5 Geowissenschaften, University of Bremen, 28334 Bremen, Germany; 10 CAGE—Centre for Arctic Gas Hydrate, Environment and Climate, University of Tromsø, 9037 Tromsø, Norway

## Introduction

As the title of the correspondence by Fossen et al.^1^ suggests, determining the age of landscape elements of the Earth surface is difficult. We thus welcome the opportunity to clarify our arguments on the contentious themes touched upon by Fredin et al.^2^ The age of landscapes has been a recurring research topic for the last century. Often, landscape ages can be deduced indirectly through morphostratigraphic correlations leading to relative chronologies. However, when working in geological contexts where a sedimentary cover is not present^1^, and the traditional geochronological tools are not suitable, not only are absolute dates of etch surface formation essentially impossible to obtain, but even relative chronologies are challenging. In an attempt to circumvent this problem, we have applied an untested methodology to date pockets of weathering products at three different sites in Scandinavia (Ivö southern Sweden, Utsira High offshore Norway, Bømlo west Norway) by K-Ar dating of illite formed authigenically during the weathering of the crystalline host rock^2^. Our results support weathering in the Late Triassic at all studied localities.

We note that Fossen et al.^1^ do not significantly question our results at two of the investigated localities (Ivö and Utsira High), where there is stratigraphical control on the age of weathering. This selective approach is questionable because the three dated sites are internally consistent with each other, of which two have independent stratigraphical control of the Triassic age of weathering. The utility of the new method should thus be discussed including the whole data set from all dated sites.

**Fig. 1:**
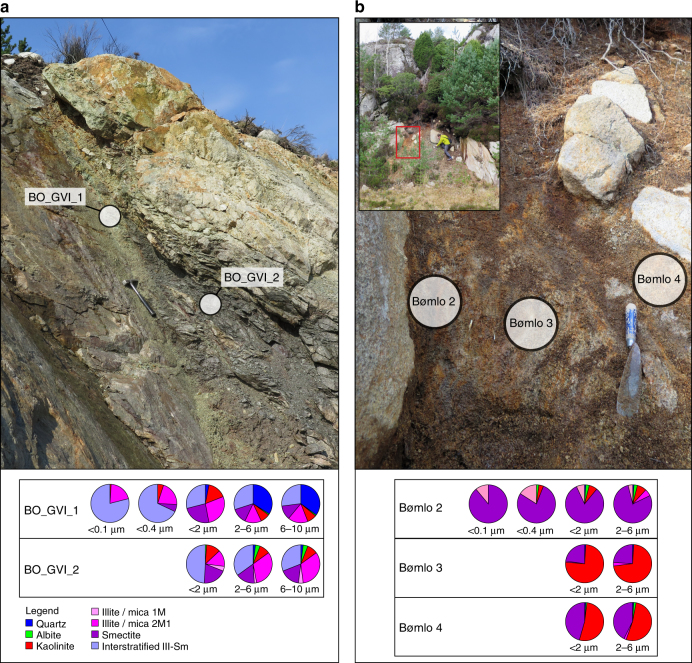
**a** Picture and XRD clay mineralogy from two fault gouge samples analysed at different grain size fractions sampled at the Goddo fault on northern Bømlo. For a detailed description of the fault anatomy and samples, see Viola et al.^4^ **b** Picture and XRD clay mineralogy from three saprolite samples analysed at different grain size fractions. The inset shows an overview of the outcrop, with red box outlining the main image (sampling site). Trowel is 20 cm long. The person (O.F.) sits at a core boulder covered with a thin veneer of glaciomarine diamicton likely of late-glacial age, which indicates that the outcrop has survived glacial overriding. The sediment-covered section was not sampled. Note that primary bedrock grain/texture is still present in the saprolite, although oxidised (rust-coloured). Grey/white portions around sample ‘Bømlo 3’ consist of kaolinite clay. For additional details, see Fredin et al.^2^

We start our rebuttal from the concluding remarks by Fossen et al.^1^, who question the saprolitic origin of the dated outcrop on Bømlo, suggesting that we might have dated a Triassic fracture. We firmly reject this possibility. Mesoscopically, the investigated outcrop lacks any evidence of a ‘structural origin’ of the dated clay-rich material. Comparison with many fractures and brittle deformation zones in the surrounding excludes that the dated illite results from synkinematic authigenic growth during faulting^3^. More telling, the detailed XRD analysis of clays in three samples from a traverse across the saprolitic outcrop documents clay assemblages that are typical for chemical weathering (Table 2, Fredin et al.^2^). We already showed that the sample closest to the fresh bedrock exhibits a less mature clay-weathering signature, whereas the sample farthest away from the fresh host rock contains a higher concentration of mature weathering products, such as kaolinite, and lower contents of immature clays such as smectite^2^. This spatially controlled mineralogical pattern is consistent with rock alteration through chemical weathering and not a faulting, fracturing or hydrothermal origin. Here, we further reinforce this interpretation by comparing the clay mineralogy of a nearby fault (the Goddo Fault, studied in detail by Viola et al.^4^) with that of the dated saprolite outcrop. The Goddo Fault phyllonitic sample BO_GVI_2 contains illite/mica and interstratified illite-smectite, with only subordinate kaolinite (Fig. 1a). The clay-rich sample BO_GVI_1 from the fault core also contains a similar clay mineralogy (Fig. 1a), but with dominant interstratified illite-smectite. In contrast, the saprolitic sample closest to the hosting fresh granodiorite (sample ‘Bømlo 2’ in Fig. 1b) is dominated by smectite with subordinate illite and kaolinite. The central portion of the saprolite outcrop (samples ‘Bømlo 3’ and ‘4’), instead contains predominant kaolinite, an end-member product of weathering. Importantly, samples from the weathering profile still preserve in situ primary mineral textures and grains from the host rock, although the rock is sufficiently altered through chemical alteration to easily disaggregate when manipulated by hand. In addition, the outcrop-bounding bedrock joints exhibit a rounded morphology consistent with the fact that weathering first attacks sharp edges, a process that produces conspicuous core boulders (‘woolsack morphology’). In summary, we remain confident that the dated samples from Bømlo are saprolitic in origin and that, therefore, the obtained ages reflect weathering.

Fossen et al.^1^ stress that available low-temperature geochronological (LTG) data in western Norway indicate that the present strandflat level was buried down to depths of >4 km in the Late Triassic and >2 km in the Early Jurassic, requiring that K-Ar ages of weathering products should be younger than any LTG data. We agree but note that available LTG data in the study area exhibit a large scatter with ages ranging from Middle Triassic to Late Jurassic^1, 5^. Furthermore, Utami^6^ reports seven apatite fission-track ages from the Bømlo area varying within very short distances between 227 and 165 Ma. In addition to this variability, we also note that important criticism by Fossen et al.^1^ are based on the age and thermal modelling (with only limited time-temperature paths; Fig. 2^1^) of one single sample (BG-113, Ksienzyk et al.^5^) from Sotra, ca. 50 km north of the sampled saprolite locality on Bømlo. Sample BG-113 suggests that sub-aerial exposure of the strandflat level on Sotra is unlikely in the Late Triassic^1, 5^. On the other hand, thermal modelling by Utami^6^ (whose results are also heterogeneous and vary from sample to sample) indicates that temperatures of 20–60 °C were locally attained in the Late Triassic, which agrees with near surface conditions and possible saprolite formation in Bømlo at that time (e.g., sample JN-06, close to the dated saprolite;^6^ Fig. 4.10 in Utami^6^). It has to be stressed that saprolite and saprock can form in a spatially heterogeneous manner down to great depths (up to several hundred meters) under extreme tropical conditions on tectonically stable cratons, implying that LTG and weathering K-Ar illite age data might converge. The kinetics of illite growth in saprolite is unknown, but is presumably geologically fast, making the system more sensitive to evolving geological processes than regional cooling/exhumation. It is likely that what is now Scandinavia was affected by severe hot-house conditions in the Late Triassic-Early Jurassic^7, 8^, and that we sampled the deepest section of a saprolite profile that might have been tens to hundreds of meters thick before subsequent stripping. We thus suggest that also LTG data from this area should be interpreted with caution and tested against independent results.

We point out that correlating denudation surfaces on and offshore using topographic profiles is controversial, as highlighted by the recent debate on palaeolandscapes in Scandinavia^9, 10^. One needs to be cautious when assigning relative landscape ages based on differences of ~5° in dip between the sub-Middle Jurassic denudation surface offshore and the current strandflat onshore, especially in the light of the local obvious post-Triassic faulting and block tilting^11^. The dipping sub-Middle Jurassic palaeosurface might well have attained its dip due to offshore faulting and differential subsidence upon sediment loading.

While the results of Fredin et al.^2^ might not fully constrain the age and complex genesis of the strandflat, the published data yield a maximum Mesozoic age for its initial formation. We conclude that the strandflat was initiated in the Mesozoic (as also suggested by Fossen et al.^1^, who wrote that the strandflat ‘may contain Mesozoic elements’), rather than completely in the Pleistocene, as indicated by early investigations^12^. We maintain that strandflat genesis at Bømlo is multi-genetic and multi-episodic, and deep weathering in the Mesozoic facilitated extensive Pleistocene erosion^13–15^.

### Data availability

The authors declare that the data supporting the findings of this study are available within the paper.
